# Editorial: Insights in inflammatory eye diseases: 2021

**DOI:** 10.3389/fopht.2022.962522

**Published:** 2022-08-05

**Authors:** Heping Xu, Narsing A. Rao

**Affiliations:** ^1^ The Wellcome-Wolfson Institute for Experimental Medicine, Queen’s University Belfast, Belfast, United Kingdom; ^2^ Aier Institute of Optometry and Vision Science, Changsha, China; ^3^ Department of Ophthalmology, University of Southern California (USC)-Roski Eye Institute, Los Angeles, CA, United States; ^4^ Keck School of Medicine, University of Southern California, Los Angeles, CA, United States

**Keywords:** inflammation, immune privilege, infection, retina, microbiota, ocular surface, gentic

Inflammation is a protective response of the immune system against endogenous and exogenous insults. The protection, however, is not without costs. Activated immune cells and the inflammatory mediators released by them can cause collateral damage leading to inflammatory diseases. To minimize the collateral damage, tissues have evolved immune regulatory machinery. The tissues are not simply passive recipients of immune protection but are active participants in their own defense (1). When tissues suffer from an insult, first, they will decide whether to call for assistance from the immune system. They will then decide which immune cells should be summoned and determine their functions (1). The type of insults, the microenvironment of the affected tissue, genetics of the hosts, and the external environments the hosts are exposed to, etc. are the determinants of the inflammatory response. The eye has an extremely high level of control over its immune response and is an “immune-privileged” organ. The core function of the eye is to acquire vision, and this requires the eye to maintain the fine structure and function of both the optical system (cornea, lens, iris, and vitreous body) and the light-sensing system (retina and the retinal pigment epithelial cells). To minimize inflammation-mediated collateral damage, the eye has developed a sophisticated protective mechanism over billions of years of evolution. This includes the physical barrier (e.g., blood ocular barrier, lack of lymphatic system inside the eye), the chemical and immunological barrier (e.g., membrane-bind and soluble forms of immune regulators expressed or released by ocular cells), and systemic immune regulation (e.g., anterior chamber-associated immune deviation, ACAID) (2–4). Despite the high level of control over its immune response, inflammation can still be dysregulated and inflammatory eye diseases are the major cause of blindness worldwide. We are pleased to present a comprehensive collection of original research and review articles in this Research Topic, “Insights in Inflammatory Eye Diseases: 2021,” which discusses the mechanisms of ocular immune dysregulation and novel insights into inflammatory eye diseases.

Genetic predisposition and epigenetic modification can alter the susceptibility of tissue to endogenous and exogenous insults as well as influence the behavior of immune cells during inflammation. Genetic risk factors are involved in many inflammatory eye diseases, among which Behcet’s disease (BD) is a typical example. Gao et al. reviewed the most recent advancements in the research of the genetics and epigenetics of BD, including the genetic variants identified from genome-wide association studies (GWAS) and candidate-association studies. The article also summarized recent advances in BD-related epigenetic modifications including DNA methylation and histone modification.

Environmental factors are one of the key contributors to human diseases. The air we breathe, the food we eat, and the water we drink all influence our health. One of the ways that the environment affects us is through microbiota. Gut microbiota contributes directly and indirectly to human health and disease (5, 6) including the eye (7). Jayasudha et al. presented an elegant study that compared the ocular surface microbiota in bacterial keratitis patients with those in healthy individuals. The study uncovered the ocular surface’s dysbiotic changes as a potential mechanism of exacerbated inflammation in bacterial keratitis.

Among various ocular tissues, the neuronal retina is most vulnerable to inflammatory insults; therefore, it has the highest levels of protection against infection. However, increasing evidence suggests that many “non-infectious” intraocular inflammations are, indeed, initiated by infectious agents. Ocular tuberculosis is a typical example of inflammatory eye disease where, in many cases, microbiological evidence could not be identified in ocular samples. Basu presented an interesting review article entitled “Absence of Evidence as The Evidence of Absence: The Curious Case of Latent Infection Causing Ocular Tuberculosis” in this Research Topic of *Insights into Inflammatory Eye Diseases*. He suggested that intraocular inflammation in ocular TB is not merely an immunologic response to bacilli, but an active tuberculosis infection. The author further discussed the reason for the frequent absence of microbiological evidence in the eye in ocular TB and the diagnostic hierarchy to arrive at the diagnosis of this infectious uveitis entity. Forrester et al. offered a further insightful explanation of the latent form of infectious intraocular inflammation in their review article entitled “Immune privilege furnishes a niche for latent infection.” Based on the evidence from studies of the central nervous system (CNS, including the brain and retina), the authors presented a view that immune privilege in the CNS may be permissive for latent infection and allow the eye and the brain to act as a reservoir of pathogens, which often remain undetected for the lifetime of the host, but in states of immune deficiency may be activated to cause sight- and life-threatening inflammation.

One of the challenges in developing safe and effective therapies for inflammatory eye diseases is the lack of understanding of exactly how inflammation leads to ocular tissue functional alteration. Two articles address this issue, one (Garriz et al.) focused on the ocular surface and the other (Shao et al.) focused on the posterior segment of the eye and the retina. Garriz et al. investigated the role of proinflammatory cytokines (IL-1β, IFN-γ, and TNF-α) on lacrimal gland myoepithelial cell contraction. Their study suggests that inflammatory cytokines can affect lacrimal gland myoepithelial cell contractile ability and reduce tear secretion, which may contribute to Sjogren’s syndrome-related dry eye disease. Shao et al. investigated the effect of INF-γ in experimental autoimmune uveitis (EAU). They found that IFN-γ promoted Th17 response at the early stages of EAU but suppressed Th17 response at the late stages of EAU. They also found that IFN-γ could modulate γδ T-cell activation. Their data suggest that IFN-γ may function as an important immunomodulatory molecule in EAU. Data presented in the two articles suggest inflammatory cytokines may affect ocular tissue function differently in different parts of the eye and at different stages of the disease.

The six articles in this Research Topic: *Insights in Inflammatory Eye Diseases: 2021* provide a broad discussion of recent insights into inflammatory eye diseases that covers the cause (genetic, environmental factors, and retinal immune regulation) of ocular immune dysregulation as well as the mechanism of inflammation-mediated ocular damage. We believe the collections are informative for basic researchers, clinical scientists, and patients who are affected by inflammatory eye diseases.

## Author contributions

HX wrote the manuscript, NR revised the manuscript. All authors read and approved the submitted version of the manuscript.

## Funding

The work of HX's lab is funded by Fight for Sight (UK) (5105/5106; 5057/5018), the European Union's Horizon 2020 research and innovation program under the Marie Skłodowska-Curie (No 722717), Medical Research Council (MR/W004681/1), Aier Eye Hospital Group Research Funds and Hunan Science & Technology Association (2018KX001; 2018RS3123).

## Conflict of interest

Author HX has sponsored research agreements with Boehringer Ingelheim and consultant agreements with F. Hoffmann-La Roche Ltd and Innostellar Biotherapeutics Co.

The remaining author declares that the research was conducted in the absence of any commercial or financial relationships that could be construed as a potential conflict of interest.

## Publisher’s note

All claims expressed in this article are solely those of the authors and do not necessarily represent those of their affiliated organizations, or those of the publisher, the editors and the reviewers. Any product that may be evaluated in this article, or claim that may be made by its manufacturer, is not guaranteed or endorsed by the publisher.
